# Learning health systems using data to drive healthcare improvement and impact: a systematic review

**DOI:** 10.1186/s12913-021-06215-8

**Published:** 2021-03-05

**Authors:** Joanne Enticott, Alison Johnson, Helena Teede

**Affiliations:** 1 Monash Centre for Health Research and Implementation, Monash University, 43-51 Kanooka Grove, Clayton, VIC 3168 Australia; 2 Monash Partners Academic Health Science Centre, 43-51 Kanooka Grove, Clayton, VIC 3168 Australia

**Keywords:** Health services research, Learning health systems, Health data hubs, Digital health

## Abstract

**Background:**

The transition to electronic health records offers the potential for big data to drive the next frontier in healthcare improvement. Yet there are multiple barriers to harnessing the power of data. The Learning Health System (LHS) has emerged as a model to overcome these barriers, yet there remains limited evidence of impact on delivery or outcomes of healthcare.

**Objective:**

To gather evidence on the effects of LHS data hubs or aligned models that use data to deliver healthcare improvement and impact. Any reported impact on the process, delivery or outcomes of healthcare was captured.

**Methods:**

Systematic review from CINAHL, EMBASE, MEDLINE, Medline in-process and Web of Science PubMed databases, using learning health system, data hub, data-driven, ehealth, informatics, collaborations, partnerships, and translation terms. English-language, peer-reviewed literature published between January 2014 and Sept 2019 was captured, supplemented by a grey literature search. Eligibility criteria included studies of LHS data hubs that reported research translation leading to health impact.

**Results:**

Overall, 1076 titles were identified, with 43 eligible studies, across 23 LHS environments. Most LHS environments were in the United States (*n* = 18) with others in Canada, UK, Sweden and Australia/NZ. Five (21.7%) produced medium-high level of evidence, which were peer-reviewed publications.

**Conclusions:**

LHS environments are producing impact across multiple continents and settings.

**Supplementary Information:**

The online version contains supplementary material available at 10.1186/s12913-021-06215-8.

## Introduction

The transition to digital health including electronic medical records (EMR) is creating the opportunity and expectation that big data will drive the next frontier of healthcare improvement and transformation. However, there are many barriers to data driven healthcare improvement and many approaches have emerged including the Learning Health System (LHS). A LHS was originally defined by the Institute of Medicine as a broader system in which “science, informatics, incentives, and culture are aligned for continuous improvement and innovation, with best practices seamlessly embedded in the delivery process and new knowledge captured as an integral by-product of the delivery experience” [1, 2]. LHS models embed data-driven research within healthcare, integrating infrastructure and multidisciplinary expertise to deliver improved health [1, 3–6], via improved access to, and increase use of data to inform clinical decision making [6, 7]. LHS apply cyclical processes to turn practice into data, analyse it to generate new knowledge and then implement this knowledge into practice in an ongoing and timely manner to support near-time improved healthcare and outcomes. A LHS is service-led and community-led to ensure relevant issues are addressed (relevant to clinicians and patients). In a LHS, higher quality, safer, more efficient care is anticipated [8–10], and health delivery organizations become better places to work [8]. The LHS in principle offers a data-driven approach to develop healthcare improvement initiatives incorporating cyclical systems and processes, expertise and resources within a central data hub [6, 11].

The LHS was prioritised in a national process to establish joint priorities using a modified Delphi process and nominal group technique. Stakeholders involved in the priority setting process included representatives from national health data organisations, government agencies, consumers and all centres from the Australian Health Research Alliance [7]. However, only a minority of healthcare organisations worldwide function as a LHS, according to only 15% of global healthcare leaders who described their organisations as adept in data-driven processes to support informed point of care decisions [12]. Evidence of and learnings from functioning LHS that have improved healthcare, are now vital to accelerate adoption and enable digital medicine to iteratively generate new knowledge and shape healthcare moving forward.

A prior 2016 systematic review examined impacts arising from a LHS and identified five papers from four LHS environments all in the United States [3]. The literature in LHS is growing with ten citations in 2007 peer-reviewed literature and over 1000 in 2017 [4]. Yet this field has been plagued by a lack of consistent terminology including data hubs, living labs, incubator, innovation or informatics hubs, learning networks, learning laboratories, community-clinician participatory data healthcare research, data driven improvement initiatives, interventional informatics, practice based data networks, circular data-driven healthcare and the LHS (refs). The LHS “community’ is also fragmented, with a lack of awareness of other’s work and limited shared learnings, leading to duplication and the lack of a critical mass of researchers and thought leaders to address barriers to adoption, maintenance, reach and sustainability [4].

For a LHS to generate new knowledge and shape the delivery and transformation of healthcare, arguably these should be health service and community-led to ensure priority areas for clinicians and patients are addressed in ways that are relevant to local settings, resources and health care systems. However, despite the availability of big data from health care, little is known about how to create effective, sustainable and service-led LHS environments that stimulate partnerships across academic, clinician, community, primary care and industry stakeholders to utilise data to iteratively to achieve better health outcomes and service improvements. To address this, an effort is underway to develop a framework for a national network of sustainable LHS data hubs across Australia. A co-design process was applied including national stakeholder engagement, governance, semi-structured interviews with international and national stakeholders and workshops were completed. To inform this work, we aimed to complete a systematic review on LHS (or similar entities with alternative names) facilitation of data-driven healthcare improvement and impact. Any reported impact on the process, delivery or outcomes of healthcare was captured. This addresses a key knowledge gap on the impacts of LHS [13].

Although some literature identifies a LHS having operational precision medicine capabilities at point of care [4], we took a broader definition which was informed by stakeholder needs. We define a LHS as a system in which routine health practice data, from service delivery and patient care, can lead to iterative cycles of knowledge generation and healthcare improvement.

## Method

We followed the PRISMA (Preferred Reporting Items for Systematic reviews and Meta-Analyses) statement for conducting and reporting a systematic review [14]. This review was registered in PROSPERO (CRD42020153319).

A systematic search of both academic and grey literature identified available publications that met the inclusion criteria. To ensure a comprehensive representation of the literature, we included publications that used qualitative, quantitative, mixed and case study methodologies, and cross-sectional, cohort, experimental and observational designs. The review processes are provided below. Also see the section describing author contributions for further details of who undertook the review tasks.

### Data sources and search strategy

An electronic search was conducted of Scopus, CINAHL, EMBASE, MEDLINE, Medline in-process and Web of Science databases, in March 2019 and again to check for any new publications in September 2019. Abstracts and publications were imported into and managed within EndNote × 8 (https://endnote.com/wp-content/uploads/m/pdf/en-x8-qrg-windows.pdf). A library scientist (AY) guided the search strategy, using a combination of keywords and wildcards, with appropriate truncations tailored for each database. The code used to search each electronic database are shown in Appendix 1. Publications were limited to English language and the past 5 years (2014 – present) to maintain currency as an emerging field and update the last systematic review in 2016 [3]. To ensure a comprehensive representation of the literature, qualitative, quantitative, mixed methods and case study methodologies, and cross-sectional, cohort, experimental and observational designs were included. To identify any additional articles, the reference lists of included publications were searched manually.

### Study selection

Titles and abstracts of retrieved publications were screened independently by two reviewers (JE, ACJ) to identify publications that potentially met the inclusion criteria. Full text of potential eligible publications were retrieved and independently assessed for inclusion by the same authors. When discrepancies occurred, consensus was reached through discussion between reviewers.

### Inclusion criteria

Inclusion criteria included publications that described an operating LHS (research focused on LHS data analysed) and translation of research evidence generated from LHS data into healthcare improvement. Table 1 outline the publication types about LHS, and indicates the type sought in this review. Appendix 2 shows the template used to determine eligibility. Exclusion criteria included post hoc analyses using registry or survey data, animal research, poster abstracts, basic research, non-English language articles, publications before 2014 and research in a low or middle income country using World Bank Atlas classifications [15]. The review focused on high income countries, as LHS require rapidly developing and sophisticated data driven systems which need advanced infrastructure, skills and systems, generally not yet established in low or middle income countries. Articles that were not reporting primary studies (e.g. reviews, editorials, commentaries, opinion pieces) were also excluded but, if relevant, reference lists were checked for additional eligible articles.

**Table 1:**
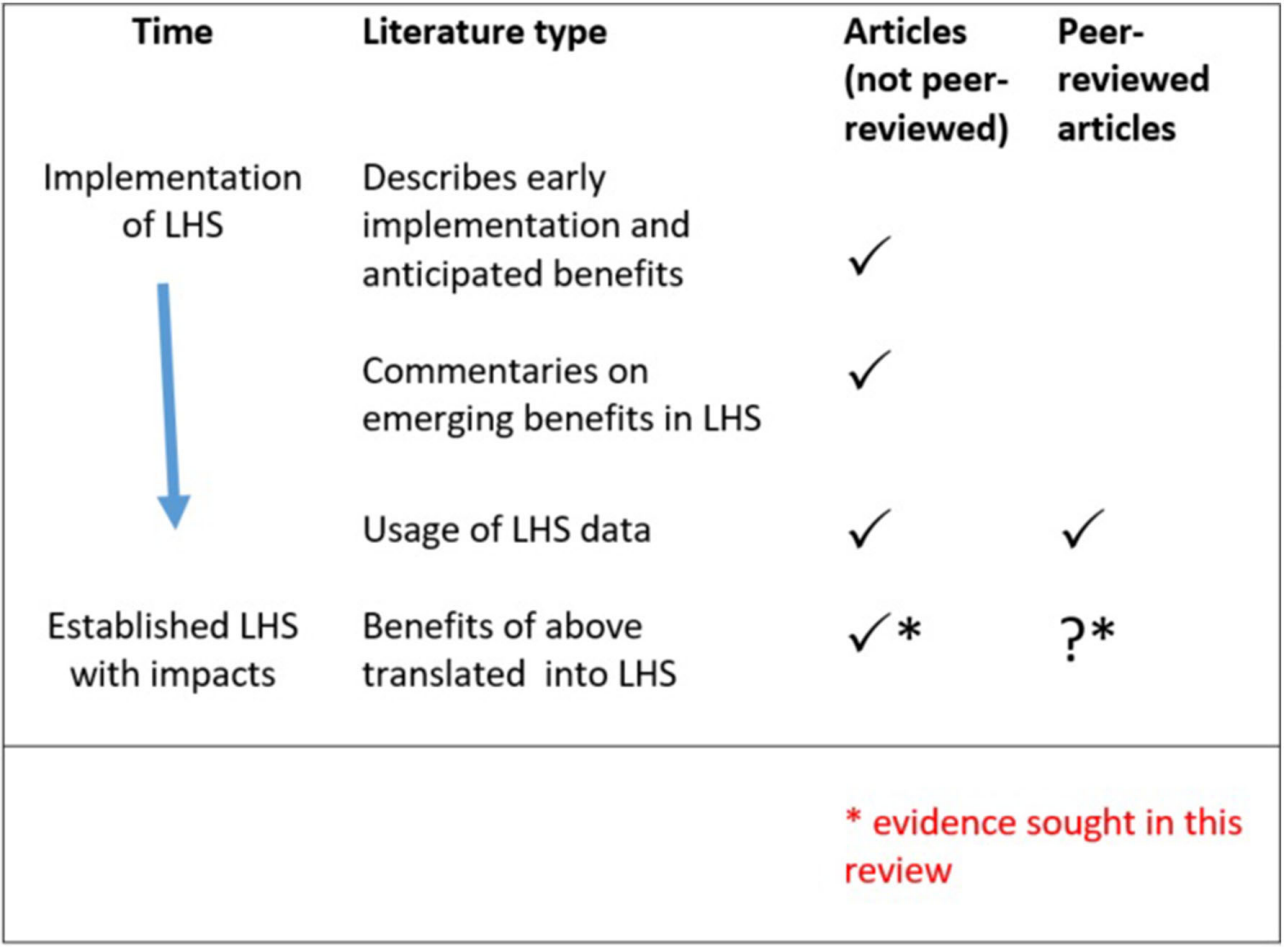
Journey of a LHS and evidence of impact in the literature. Ticks indicate literature types readily available at the time of writing. *This review seeks to identify the evidence and research translated into the LHS environment

Publications were included if they reported the following according to the Participants, Intervention, Comparator and Outcome (PICO) approach [16]):

- Participants included health providers (key and could not be nonessential or passive participants) and the setting included community and health care organisation(s) delivering services to patients;
- Interventions such as initiatives using data for healthcare improvement, new data capability embedded in health services to drive utilisation of data for the purpose of healthcare improvement, embedded data roles, knowledge mobilisation or brokering (with data as significant component), improving data capability (e.g. how to use existing data), usage of live (key and could not be nonessential or passive participants) analytics such as dashboards (e.g. by clinical staff) and data feedback mechanisms involving clinicians.
- Comparators were not essential
- Outcomes in eligible articles reported evidence of a LHS translating data-driven research into healthcare, with measurable impact at the patient or service improvement level (e.g. patient health impact measures; patient self-reported outcomes, measures of utilisation of best practice guidelines, clinical variation, access to integrated service systems utilising data and evidence of translation into practice.

### Grey literature

Peer-reviewed literature was supplemented with a search of the grey literature using a general Internet search with Google and Google Scholar. In addition, we asked the investigators and stakeholders (*n* = 26, identified as working and providing leadership in data hubs, health care services and/or research and interviewed in a related study about LHS [17]) to identify relevant sources of literature in the form of websites, newsletters, online or print reports, annual reports, research or quality assurance reports, any persons that had established a data hub, and any another relevant contact person. Free text searching used the same search terms, and inclusion and exclusion criteria noted above. The search of the grey literature ended Sept 2, 2019.

### Data extraction and quality assessment

One author (JE) extracted data from the included publications and identified the level of evidence. Publications with heterogeneous study designs were anticipated, therefore the GRADE Approach was applied to assess overall quality of evidence based on the study design [18]. In the GRADE approach, randomized trials without important limitations provide high quality evidence, and observational studies without special strengths or important limitations provide low quality evidence. GRADE recommends that design factors such as ‘concurrent controls’ can improve the quality of evidence, therefore, studies with concurrent controls without important limitations were assessed as providing medium quality evidence. We also assigned a level of evidence as ‘0’ if publications could not be assessed because it was (a) a peer-review publication that stated the translational benefits of a LHS but provided no objective evidence as no values were provided, or (b) a non-peer reviewed article i.e. grey literature.

### Data synthesis & analysis

Due to the heterogeneity of interventions, study designs and outcomes, narrative synthesis methods were used. Narrative synthesis collates the collective findings into a coherent, textual narrative, and is appropriate when the review question dictates the inclusion of a wide range of research designs, producing qualitative and/or quantitative findings for which other approaches to synthesis are inappropriate [16].

LHS impact categories were determined by authorship panel of experts and were based on the healthcare improvement and impact reported in the study. These categories were designed to be broad and inclusive, acknowledging that benefits were often noted across categories, hence the primary reported outcomes determined the final categorisation. These categories were: Benefits to patients; Benefits to clinician and patient encounters; Benefits to clinical services, organisation and system-level performance, and; Benefits to research and evidence generation.

The included studies were grouped together based on the overarching LHS concept. This was done because the review aimed to gather evidence on the effects of LHS (or similar entities with alternative names) and a LHS by design is a system level intervention that includes multiple processes and projects. This is an accepted process for reporting diverse health-related initiatives in a single peer-reviewed research publication.

## Results

**Fig. 1:**
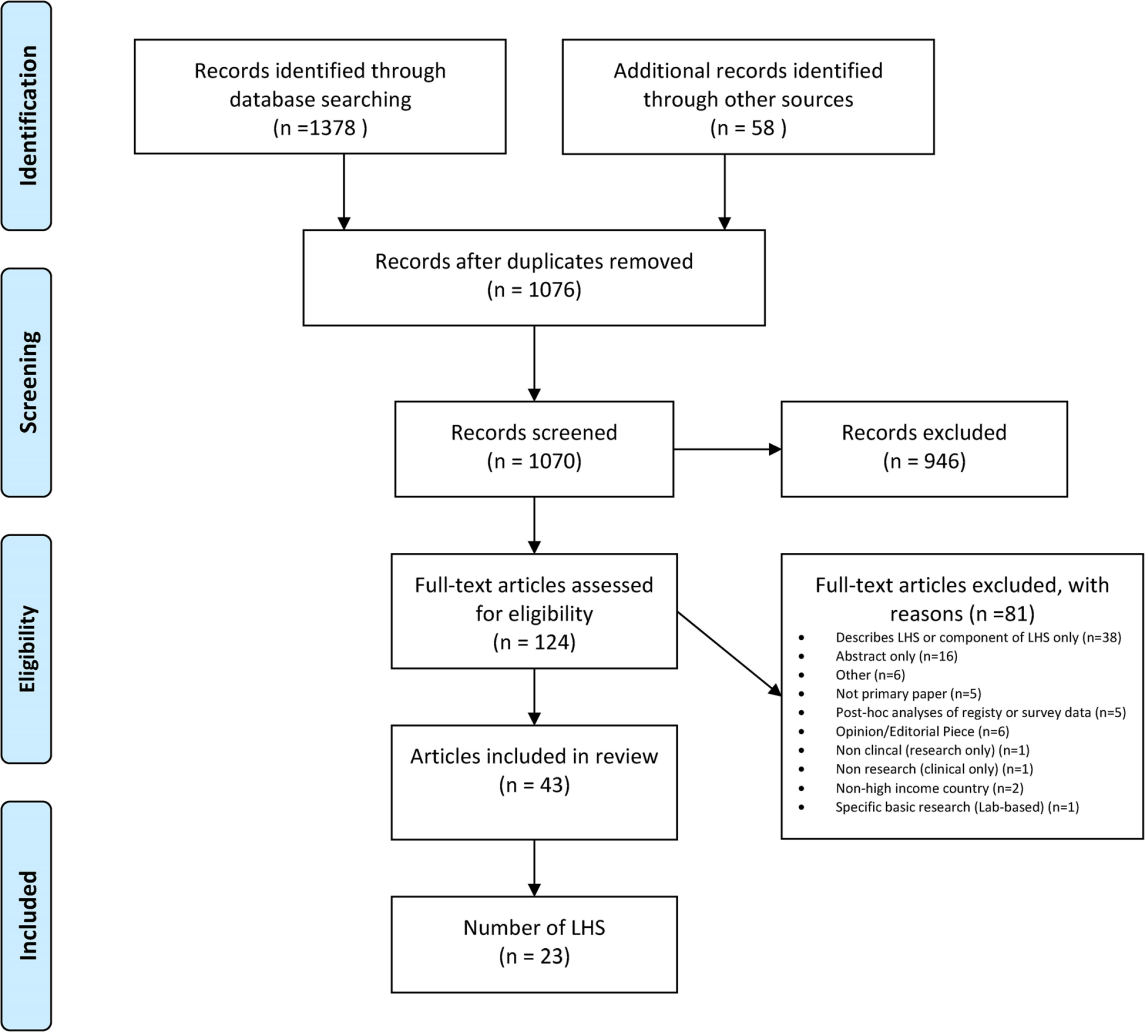
PRISMA Flow Diagram

The search identified 1076 titles after duplicate removal. Screening of titles and abstracts excluded 946 of these. The remaining 124 full-text articles were examined and a further 81 excluded. This left 43 articles meeting inclusion criteria. Overall, the bibliography database search only identified 26% (11/43) of articles and the grey search identified the remainder. Search results are summarised in the PRISMA flow diagram in Fig. 1 and in Table 2.

**Table 2:** Learning health systems with reported outcomes. *Peer-review article

	LHS name	Country (scale)	Purpose	Impact outcomes
1	ePRO Duke cancer clinics LHS [19]	United States (Local)	To build LHS infrastructure with patient-reported outcomes in EHR in a cancer clinic.	Electronic administration of distress screening, provided immediate scoring, & facilitated triage. Evaluation done on longitudinal impact of a psychosocial care program provided to patients with breast cancer. Significant improvement in distress and despair as measured by self-report questionnaires at 3 and 6 mo *
2	Surgical Care and Outcomes Assessment Program (SCOAP) [20]	United States (Regional)	A peer-to-peer surgeon collaborative that create & track process of care metrics, and interventions to correct under performance.	Multisite benchmarking led to decreased percentage of postoperative complications (17.7–9.6%), increased use of imaging, testing, blood glucose checks. *
3	ImproveCareNow Chronic Care Network [21, 22]	United States (National)	To empower clinicians, researchers, parents and youth to learn and continuously improve care and outcomes for chronic diseases like inflammatory bowel disease.	Improvements in the remission rates Time savings of 7 min per patient visit due to automatic data transfer. Staff using & benefiting from a learning exchange platform. *
4	NUCATS Institute LHS [22, 23]	United States (Regional)	To create a central hub supporting clinical and translational research. Dual-use model warehouse designed to serve both research and clinical needs, integrating healthcare and research.	Greater than a 2.5-fold increase from 2011 to 2013 in data requests by affiliates. * Created dashboard enabling real-time monitoring of transplant outcomes, replacing a slow manual process. Wagner et al. 2015 introduced PROMs into routine cancer care, automatically sent to patients prior to appointment, 80% (874) of pts. who read message completed questionnaires. TOPCAT study recruitment: daily reports identified potentially eligible inpatients (based on free-text, laboratory, imaging, and medication data). Northwestern became top US enrolling site. Change in care as HFpEF pts. identified in reports are now invited to a specialized clinic.
5	Ottawa Hospital Lung Cancer LHS [27, 28]	Canada (Regional)	To drive system optimization & innovation in cancer care using community of practice (cop), hub-and-spoke infrastructure, and regional steering committee. Later, to operationalize LHS thinking, developed the Ottawa Health Transformation Model (ohtm)	Compliance with provincial evidence-based clinical guidelines improved (20% increase in 2010–11 compared to 2006–07). Other improvements were standardization & implementation of regional perioperative pathways; rectal cancer surgery centralization; increased use of sentinel lymph node biopsies in breast cancer surgery; and decreased positive surgical margin rates in prostate cancer.* Lung cancer diagnosis now provided to 80% of referrals within target of 28 days. Median patient journey from referral to initial treatment decreased by 48% from 92 to 47 days.
6	PEDS-CHOIR [29]	United States (Local)	Tertiary care clinic registry to guide research and precision pain medicine in pediatric populations.	Captures patient-caregiver PROM/PREM data in real time, to augment clinical decisions and treatment delivery. * Completion rates increased after staff training, clinic flow enhancements, & conversations with patients, clinicians, and staff highlighting the benefits of personalizing & optimizing patient care over time. Completion adherence increased: - first pt. survey: 82.4 to 91.7%, - subsequent pt. surveys 17.3 to 57.6%
7	University of Alabama at Birmingham Hospital, USA [30]	United States (Local)	Collaboration between healthcare finance leaders & physicians and sharing a vision to create KPIs that pairs patient-centred outcomes with increased efficiency.	Surgeons now access KPIs (scorecards), showing performance and benchmarks. Physician committee monitors scorecards & provide feedback.* *(improved quality and savings but no figures provided)*
8	ePPOC [31]	Australia and New Zealand (National)	Chronic pain registry to guide patient clinical care and research, and to establish a benchmarking system to drive multicenter quality improvement.	Clinics submit high-quality data describing the demographic and clinical characteristics of patients. Information used in each service to assess & monitor patients and submitted to central coordinating site for analysis, reporting, and benchmarking purposes.*
9	Community Health Applied Research Network (CHARN) [32, 33]	Across four states, US (Regional)	To pool data, and promote integration of research in health centers and translate it into practice to advance evidence base for improved care in medically underserved patients.	Goal1: Fully developed an operational infrastructure to support national Patient Centred Outcomes Research. Goal2: Fully developed a consensus derived research agenda to guide the networks activities. Goal3: Fully/partially created processes to develop multimodal proposals, conduct pilot studies and carry out multimodal investigations with support. Goal4: Not accomplished, transfer of network findings into practice. Goal5: Fully developed a collaboration for bi-directional education and exchange of ideas, information and values. * (*didn’t achieve Goal 4 translation into practice, but did achieve Goal 5 of bi-directional collaborations*)
10	KPNC 30 primary care practices at 13 medical facilities across four counties [34]	Northern California, US (Regional)	To test the hypothesis that a Pre-Visit Prioritization secure email message could improve visit communication and glycemic control among patients with type 2 diabetes.	Improved measures of interaction: more patients reported preparing questions for their visit (72% vs 63%, *p* = 0.048) and being given treatment choices to consider (81% vs 73%, *p* = 0.041). Patients in both arms had similar reductions in HbA1c over the 12-month study period (0.56% ± 1.45%) with no significant differences between arms.*
11	Connected Health Cities [35] (plus final report June 2019)	UK (Regional)	To make better use of information & technology that already exists in health & social care system to improve health and ensure services are more joined-up.	Ransom 2018: 6% reduction in average no. of prescription items per person with frailty. Reduction in prescribing costs by £69 – £299 per patient year. ^Correspondence article^ Final report (Jun 2019) - Enabling Data Flows in Greater Manchester Connected Health City: 3 projects described: Opioids, Stroke, and Musculoskeletal epidemiology projects. At least 20 research projects described on webpage (NOT WITH OUTCOMES YET)
12	PCORnet® [35, 36]	US (National)	To operationalize the learning health system across several healthcare systems. Provides capacity to conduct transformative clinical research with real-world data, research capabilities, patient partnerships, and broad array of health services researchers. Disseminating and promoting the uptake of research findings is part of PCORI’s legal mandate to improve the quality and relevance of evidence available to help patients, caregivers, clinicians, employers, insurers, and policymakers make better-informed health decisions.	PCORnet Bariatric Study compared weight measures at 1, 3, and 5 years post surgery (3 types) in 44,978 patients. One type (RYGB) led to greater long term weight loss but also had highest 30-day rate of adverse events. Project results implemented in new shared decision making (SDM) model being evaluated with patients.* Information on webpage shows research (Arterburn 2018; Toh 2017*) led to a new shared decision making (SDM) model. PCORI website reports Dissemination & Implementation activities underway for 21 PCORnet projects (Limited Competition Project funded by PCORI). Peer review publications available for some at time of writing: - Wade 2017* led to *Widespread Implementation of a Patient-Centered Online Therapy for Adolescent Traumatic Brain Injury* - Lowenstein et al. 2018* led to *Implementing Patient Decision Support for Lung Cancer Screening through Tobacco Quitlines* - *Implementing Peer-Driven Care to Patients with Sleep Apnea*
13	Swedish Rheumatology Quality Register [39]	Sweden (National)	To improve the healthcare and treatment of patients with rheumatoid arthritis and other chronic diseases.	PROMs and care information are entered as routine clinical practice. Patients benefit by being involved in their own care & records. Physicians benefit by longitudinal overview of each patient including disease activity, disability and treatment. Shared decision making is facilitated. * Evaluations found patients value system for knowledge it gives them about their changing condition & symptoms over time. Data used for research purposes: e.g. peer review publications and evidence of safety from drugs (Simard 2011; Neovius 2014).*
14	University of Wisconsin Health LHS [42, 43]	United States (Regional)	To consistently deliver high value care and support continuous learning and improvement in the health system.	System-level performance improvements 2010–2015: - patient satisfaction improved 0.078 points per month and significant at *P* > .001 - pneumococcal vaccination increased 62 to 90%, and colorectal cancer screening from 69 to 81%. - Staff completing formal courses in improvement science tripled between 2012 and 15 - patient & family advisory councils increased by 83% from 90 to 165 Odds of primary care followup doubled (OR 2.0, 95% CI 1.4–2.9). Median time to followup decreased from 71 to 38 days (Bartels 2019). Implementing checklist improved delivery of Family Centered Rounds (Cox 2017).* Plus on website: - 52 Toolkits to assist implementation & change initiatives - - 397 Peer-Reviewed Articles*
15	Wound Care LHS [47, 48]	United States (National)	To integrate wound care management, quality improvement, & comparative effectiveness research by harnessing real-world data in a purpose-built electronic health record at point of care.	Centers submit clinical & quality data, enabling benchmarking across national network. * It showed patients’ contrasted strikingly with published RCT samples: real-world population are older and sicker, with common comorbidities: e.g. Pressure Ulcers healing rate 40% in 2 RCTs, and 30% in LHS.*
16	Alberta Strategic Clinical Networks [49, 50]	Canada (Regional)	To support clinically-led, evidence-informed change in Alberta’s health system. Goals of achieving best outcomes, seeking greatest value for money and engaging multidisciplinary clinicians in all aspects of the work.	Reduced inappropriate use of anti-psychotics in Long-Term Care by 20%. Improved surgical safety through effective implementation of checklist completion (was 40% and rose to 65%) and 3.5% reduction in errors Evidence based stroke care improved: results show reductions in average length of hospital stay by half, improved access to rehab, and better stroke outcomes. (no figures provided) (Noseworthy 2015) Gramlich 2017 Compliance with the evidence-based guidelines for colorectal surgery recovery *
17	Geisinger Health Systems LHS [51]	United States (Regional)	To support continuous learning and improvement across the Geisinger Health system.	Quality improvement and cost reduction programs mentioned (but no figures provided).* Website: No show rates of up to 47% reduced to 24% by using routine data to predict no show risk. High risk patients now receive a phone call prior to appointment.
18	Cystic Fibrosis Foundation Patient Registry [52]	United States (National)	To describe the cystic fibrosis population in the U.S., support epidemiological and clinical research on cystic fibrosis, and improve the quality of cystic fibrosis care.	Benchmarking and public transparency. Case-mix adjusted center results are made public on the web. Patient can access own records, which brings value to encounters for both patients and providers. Centers can access their records to track performance.*
19	Kaiser Permanente Northern California (KPNC) LHS [53, 54]	United States (National)	To support continuous learning and improvement across the KPNC health systems.	Sepsis improvement program, demonstrated significant decreases in mortality: 8.8% in 2011, 9.3% in 2012, and 7.9% in 2013 (*P* = 0.02). Decreased hospital mortality was observed primarily in patients with a heart failure and/or kidney disease history (*P* < 0.01). This corresponded to changes in care for patients with heart failure and/or kidney disease.*
20	Distributed Ambulatory Research in Therapeutics Network (DARTNet) [55]	United States (National)	To transform multi-sourced data into standardized, actionable health information that supports patient care, quality improvement, patient safety and health improvement, and collaborative learning and research.	Practices can compare their performance on many measures to each other. Jenkins 2013 significant improvements in antibiotic use in intervention compared to control practices. The clinical pathways & patient educational material (FROM Jenkins 2013) became part of the DARTNet Learning community and are used by other DARTNet associated clinical organizations.* Website promotes: learning communities for clinician, practice staff & researchers
21	Optum Labs LHS [56]	United States (National)	To improve patient care and value in the healthcare system by connecting the generation of evidence with its accelerated translation into practice and its widespread adoption into care delivery. Partnership between Optum and the Mayo Clinic.	Study compared medication management in 37,501 diabetic patients: cost was less and also longer intervals between insulin for one drug type. Findings translated into guidelines used by care providers.* Website information & online magazine Webpage article describes care in ED changed based on anaphylaxis data research: longer observation period & patient education provided with training in injectables. Over 150 publications linked to website.*
22	Learn From Every Patient LHS [57, 58]	United States (Local)	To integrate clinical care and research, and to use this knowledge to systematically deliver continual quality improvement in care.	Children with CP had 43% reduction in total inpatient days (*p* = 0.030 vs prior 12mo period), a 27% reduction in inpatient admissions, 30% reduction in emergency department visits (*p* = 0.001), and 29% reduction in urgent care visits (*p* = 0.046). Reductions in healthcare costs of 210% (US$7014/child) (Lowes 2017) Evidence-based change in hip screening procedure by eliminating annual screening x-rays for all CP patients based on LHS data research (Noritz 2018)*
23	IDEA4PS [59, 60]	United States (Local)	To generate, integrate, and disseminate research throughout the institution and to promulgate those findings for the greater good.	Designed, piloted and implemented the “Falls Wheel” into routine practice. A visual display for risk of a patient falling. * Cardiac monitoring improvements made, including decreased false alarms (18.8 to 9.6%, *p* < 0.001).*

The included 43 articles described translation into health impact across 23 LHS environments: 18 in USA, two in Canada, one each in UK, Sweden and Australia/NZ. LHS settings include local (5), regional (9) and national (9). At least one peer-reviewed article was available for each of the 23 LHS except one; *Connected Health Cities* in the UK, only reported in the grey literature with a correspondence article [35] and a final report [61]. This LHS also reported at least 20 research projects on a webpage, but not all had achieved outcomes at the time of writing (Table 2).

The remaining 41 articles were peer-reviewed. These comprised quantitative (*n* = 33), qualitative (*n* = 2), and mixed-method (*n* = 2) studies as well as (*n* = 4) publications that stated improvements but no figures were provided (and therefore assigned ‘0’ level of evidence in this review) [30, 40, 51, 56]. Five quantitative studies included a control group and were randomised controlled trials [23, 34, 36, 37, 43]. Another was a comparative study with concurrent controls [57]. Twenty-seven publications used uncontrolled quantitative approaches, predominantly reporting observational data from registries or electronic medical records (EMRs).

These 23 LHS environments can be categorised as:

- nine real-world data enabled: electronic health record and/or linked data [19, 23–25, 34, 35, 37–39, 51, 53–55, 57, 58, 62]
- six built around clinical registries [21, 22, 29, 31, 40–42, 47, 48, 52]
- four community of practice networks [20, 27, 28, 32, 33, 49, 50]
- two academic health centre initiated [43–46, 59, 60]
- one finance staff and physicians/surgeons collaboration [30]
- one commercial operation [56]

Most LHS in this review were enabled by digital data gathered from EMR’s using analytic techniques to translate data to generate new knolwedge and improve clinical or service performance [19, 23–25, 34, 35, 37–39, 51, 53–55, 57, 58, 62]). Other LHS were built around clinical registries housing uniformly collected data used to describe populations with specific diseases or characteristics and monitor their outcomes such as the registers used by ImprovingCareNow [21, 22], the Swedish Rheumatology Society [40–42] or the national Cystic Fibrosis Foundation in the United States [52].

Some LHS were initiated by services creating a community of practice particularly when linking smaller site to other sites to share learnings and expand data pools [20, 27, 28, 32, 33, 49, 50, 63]. One LHS, Optum Labs, was a commercial operation [56] collaborating with an academic partner, Mayo Clinic.

There were 14 service-led LHS identified in this review [19, 20, 23–25, 27, 28, 30, 32–35, 37–39, 49–51, 53–55, 57, 58, 62]. The service-led LHS in this review had been initiated and enabled because of newly implemented digital health data and analytic techniques (e.g. [19, 23–25, 34, 35, 37–39, 51, 53–55, 57, 58, 62]), as well as the creation of new community of practice networks [20, 27, 28, 32, 33, 49, 50]. Another service-led LHS was initiated by finance leaders in the hospital establishing a respectful and valued collaboration with the physicians/surgeons, and both groups drove the LHS to create more efficient and better surgical outcomes [30]. Improving patient access and interaction with information was key to improving patient experience and outcomes in one service-led LHS [51].

### Benefits to patients

Benefits achieved for patients were largely due to better evidence based care provided because of site/clinician benchmarking and individual patient record longitudinally tracking care and outcomes readily available at point of care. Examples of patient benefits included identifying distress and despair in cancer patients [19], decreasing postoperative complications (17.7–9.6%) [20], increasing patients in remission [21], shorter waits for lung cancer treatment commencement after referral (median 92 reducing to 47 days) [28], and reductions in polypharmacy by 6% [35].

Identifying distress was achieved in in ambulatory cancer care patients by electronically sending questionnaires prior to a visit, responses were automatically integrated into the patient EMR, and clinicians notified of clinically elevated symptoms through messages, which then facilitated referral to psychosocial and supportive care. Psychosocial concerns were reported by 34%; common psychosocial needs were information on advance directives (16%), support with managing stress (15%), information on financial resources (11%), coping with cancer diagnosis (10%), information on support groups (9%) and 25% indicated that they would like to be contacted by a health educator for assistance finding health-related information [25].

### Benefits to clinician and patient encounters

Some LHS enabled patients to track and self-manage their condition, and enable quicker and more evidence-informed decisions for clinical practice, public reporting, and research as well as for clinical process improvement [19, 42]. For example, the LHS Swedish Rheumatology Registry enables a patient to record symptoms, health status, and quality of life directly into their EMR before a clinical encounter. Patients access their own EMR at a clinic using a computer tablet or at home via a patient internet portal. The system combines these data with other data (clinical examinations and laboratory results) to give a graphical display of the patient’s health status, as well as a time graph of trends in the person’s health and treatment. The patient and clinician can view this together, or separately, and this helps the patient and clinician to partner to optimize health. Data was also exported to the national registry, enabling research to contribute to improving patient population health. Evaluations have found that patients greatly value this system for the knowledge it gives them about their changing condition and symptoms over time [42, 64].

### Benefits to clinical services, organisation and system-level performance

Benefits to service delivery were also evident e.g. time savings of seven minutes per patient visit due to automatic data transfer [22], compliance with evidence-based clinical guidelines improved by 20% [28], pneumococcal vaccination increased 62 to 90% and colorectal cancer screening from 69 to 81% [46].

An essential component of a LHS is a collaborative platform that provides connectivity across silos, organizations, and professions. Automated reports using the data from the entire LHS led to the efficient identification of patients for standardised care, specialised care, follow-up or clinical trials [21, 27, 28]. Collection of information directly from patients before the clinical encounter can improve time efficiencies, and create PROMs (patient reported outcome measures) that are saved within the EMR that enable longitudinal tracking of individual patient outcomes and aggregated research [19, 42].

Data architecture appears to be trending away from the traditional relational database and towards a hybridization of big data and high performance computing. This is driven by the differing data sources held at different sites that can be linked for the purpose of analysis (ref), or aggregated versions compared as benchmarks (ref). Benchmarking site performance can now be easily provided using aggregrated data from each site, and it has the advantage that no individual information is released. Aggregated benchmarking comparisions between clinics/services was reported to lead to subtansitial benefits in the six LHS built around clinical registers [21, 22, 29, 31, 40–42, 47, 48, 52]. The Cystic Fibrosis Foundation attributes publishing of clinic performance on a public website as an important driver for greater standardisation and implementation of evidence based care in routine practice [52].

The two LHS initiated by academic health centres had produced publications about implementation issues to develop a system-wide LHS. [46, 59] These publications acknowledged the premise of the LHS was embraced and theoretically endorsed for years, but the translation of the LHS approach and implementation into healthcare was a difficult and long undertaking. They then went on to describe longterm (> 5 years) system-level performance improvements resulting in multiple domains: patient satisfaction, population health screenings, improvement education, and patient engagement [43–46, 59, 60]. They both proposed that their experience in developing a large healthcare setting into a LHS can be applied to other health systems that wrestle with making system-level change when existing cultures, structures, and processes vary.

### Benefits to research and evidence generation

LHS models include the ability to augment participation in pragmatic real-world trials, comparison effectiveness trials, identify adverse drug effects, and follow data-driven guidelines. Efficient data extraction can directly facilitate evaluation of improvement efforts and can be used to collect data from clinical trials with reduced patient, health service and research team burden.

### Quality assessment of publications

Level of evidence for included publications are shown in Table 3. Level of evidence was assessed as high for the five RCT publications [23, 34, 36, 37, 43] and medium for one comparative study with concurrent controls [57]. A low level of evidence was assigned to the 27 publications reporting observational data from registries or EMR. Five (21.7%) of the LHS environments produced medium-high level of evidence peer-reviewed publications. These five LHS were all in the United States: three regional and two national. No evidence (lowest rating) was assigned to six articles that could not be adequately assessed for level of evidence because four were peer-review publications stating translational benefits of a LHS but no figures provided [30, 35, 40, 51, 56, 61], and two were grey literature [35, 61]. The two mixed method studies were assessed as providing low level of evidence, based on the assessment of the quantitative components. The two qualitative studies were not assessed.

**Table 3:** Level of evidence as classified by the study design for the publications included in this review. A level of evidence as ‘0’ was assigned if article could not be assessed because it was (a) a peer-review publication that stated translational benefits of the LHS but no figures provided, or (b) it was a non-peer reviewed article i.e. grey literature

	LHS		Study	GRADE Level of Evidence	Notes
1	ePRO Duke cancer clinics LHS	[19]	Observational, case series pre/post outcomes	low	
2	Surgical Care and Outcomes Assessment Program (SCOAP)	[20]	Observational, case series	low	
3	ImproveCareNow Chronic Care Network	[21]	Observational, case series	low	
		[22]	Qualitative case evaluation, examining LHS user interaction, technology, content management, and community engagement	*?*	*Qualitative evaluation*
4	NUCATS Institute LHS	[24]	Observational, case series	low	
		[25]	Observational, case series	low	
		[23]	RCT	high	
		[26]	Observational, case series	low	
5	Ottawa Hospital Lung Cancer LHS	[28]	Observational, case series pre/post outcomes	low	
		[27]	Observational, case series pre/post outcomes	low	
6	PEDS-CHOIR	[29]	Observational, cross-sectional cases	low	
7	University of Alabama at Birmingham Hospital, USA	[30]	Improvements stated, no figures provided	*0*	*No figures provided*
8	ePPOC	[31]	Observational, case series	low	
9	Community Health Applied Research Network (CHARN)	[33]	Qualitative evaluation of the adoption, expansion, and diffusion of the national LHS model	*?*	*Qualitative evaluation*
		[32]	Observational, cross-sectional cases, other improvements stated but no figures reported	low	
10	KPNC 30 primary care practices at 13 medical facilities across four counties	[34]	Pragmatic, provider-randomized, multi-site clinical trial	high	
11	Connected Health Cities	[35]	Correspondence article in journal	*0*	*Grey literature*
		[61]	Final report, observational, case studies reported	*0*	*Grey literature*
12	PCORnet®	[38]	Interrupted time series without parallel control	low	
		[39]	Two of more single arms	low	
		[36]	RCT, large multisite	high	
		[37]	RCT, large multisite	high	
13	Swedish Rheumatology Quality Register	[40]	Improvements stated, no figures provided	0	*No figures provided*
		[42]	Observational, 3 case descriptions provided	low	
		[41]	Observational, case series	low	
14	University of Wisconsin Health LHS	[46]	Observational, case series	low	
		[44]	Interrupted time series without parallel control	low	
		[43]	Cluster RCT, 4 sites	high	
		[45]	Observational, case series pre/post outcomes	low	
15	Wound Care LHS	[48]	Observational, case series	low	
		[47]	Observational, case series	low	
16	Alberta Strategic Clinical Networks	[50]	Observational, case series pre/post outcomes	low	
		[49]	Observational, case series pre/post outcomes. Thematic analysis on documents & interviews	low	*Mixed-methods*
17	Geisinger Health Systems LHS	[51]	Improvements stated, no figures provided	0	*No figures provided*
18	Cystic Fibrosis Foundation Patient Registry	[52]	Observational, case series	low	
19	Kaiser Permanente Northern California	[53]	Interrupted time series without parallel control	low	
		[54]	Observational, case series pre/post outcomes	low	
20	Distributed Ambulatory Research in Therapeutics Network (DARTNet)	[55]	Observational, case series	low	
21	Optum Labs LHS	[56]	Improvements stated, no figures provided	0	*No figures provided*
		[57]	Comparative study with concurrent controls	medium	
		[58]	Observational, case series	low	
23	IDEA4PS	[59]	Observational, case series	low	
		[60]	Observational, case series pre/post outcomes. Ethnography and interviews	low	*Mixed-methods*

Level of evidence was not determined for qualitative approaches and instead notes on the qualitative approach are provided

## Discussion

With the flood gates of health data now open, there are clear opportunities to turn practice into data, data into new knowledge and knowledge into improved practice, however there is limited evidence of effective systems level approaches and processes to deliver on these opportunities. This systematic review and narrative evidence synthesis shows that LHS environments are increasing with demonstrated health benefits across multiple continents and a range of settings. LHS built on electronic medical records and/or linked data, clinical registers, community of practice networks, academic health science centre partnerships, medical collaboration or commercial operations. Benefits were noted in patient self-management, evidence-based clinician care, clinical organisation or system-level performance and in research. Core features of LHS included having strong partnerships, generating a shared vision across stakeholders, having agreed principles and governance, implemented systems and processes to enable iterative sustainable improvement, and longitudinally benchmarking and patient tracking with feedback to frontline patients, clinicians and health services. System-level performance improvements were evident in multiple domains: patient satisfaction, population health screenings, improvement education, and patient engagement. Quality was variable and limitations included poor alignment of terminology.

The novelty of this systematic review compared to past LHS reviews lies in the research aim, inclusion criteria and the systematic methods. This resulted in included studies that needed to report impact on the process, delivery or outcomes of healthcare arising from the LHS. Unlike a recent white paper [65] and other LHS reviews [4, 6, 13, 65], here papers were excluded if they described a LHS or usage of data in a LHS, without reporting impact. As noted by Foley and Vale (2017) [13], further research to evaluate the impacts of LHSs is needed, and we sought to advance this in the current systematic review.

The previous systematic review on LHS in 2016 identified only five publications from four LHS environments, all within the United States [3]. Here we have identified 43 studies from 23 LHS environments across continents and settings. We note that significant future evidence is anticipated with LHS such as the “Connected Cities UK” noting over 20 projects in the grey literature, that are yet to report. The USA has also invested $8 million annually since 2018 to build workforce capacity across 10 institutions to establish a sustainable corps of learning-health-system researchers [66]. A dedicated journal was established in 2017 to advance the interdisciplinary area of learning health systems, to enable continuous rapid healthcare improvement and transformation of organisational practices. Yet here many studies were identified through the grey literature search and reference list checks and inconsistent terminology remains a key barrier to progress. Moving towards consistent terminology would enable the capture and sharing of learnings on how to design, implement and sustain the complex system level interventions needed in an effective LHS. Furthermore learning from these large programs underway in the US and the UK will yield more learnings on effective LHS models.

The underpinnings of the LHS included electronic medical records and/or linked data and clinical registers a core data sets. Organisational structures included community of practice networks, academic health science centre partnerships, medical collaboration and commercial operations. The LHS environments producing impact identified in this review show that LHS were not homogenous entitiies and can have a range of operational scale and orginate from different origins. For example, we identified *n* = 5 local (eg. hospital), *n* = 9 regional (eg. networks of healthcare providers) and *n* = 9 national (eg. linked services in a country). Similarly, Menear et al. (2019) noted that the LHS could differ in scale, operating locally, regionally, nationally or even internationally [5], and implied that local or regional LHSs can evolve alongside or within broader LHSs, with linkages between LHSs or between actors at various system levels [5]. Origins ranged from clinical registries to new clinician community of practices, which then grew into the operational LHS environments.

Benefits noted included patient self-management, evidence-based clinician care, clinical organisation or system-level performance and benefits in research. To have direct health impact, a LHS must provide timely access to data as well as analysis of that data. Access to integrated real-world data is often impeded by governance and regulatory systems as well as technical, quality and interoperability issues. This review showed that these issues can be addressed within the LHS continuous improvement process, supported by strategies including natural language processing to improve data quality. The effective LHS identified in this review combined people with relevant workforce capacities and people with capabilities in analytics to make sense of the complex data arising from complex improvement cycle focuses on areas of unmet need, public interest and priorities. This was particularly evident in the service-led LHS environments including the registry-based LHS.

Core features of LHS included having strong partnerships, generating a shared vision across stakeholders, having agreed principles and governance, implemented systems and processes to enable iterative sustainable improvement, and longitudinally benchmarking and patient tracking with feedback to frontline patients, clinicians and health services. LHS environments translating data-driven evidence into clinical practice and identified in this review all confirm that a key feature to achieve this are integrated multidisciplinary teams of frontline clinicians, researchers and community members, embedded in healthcare. This is critical for the purpose of using data from clinical encounters and other sources to generate new knowledge to continuously inform and improve health decision making and practice. This is commensurate with the views of the LHS literature dating back to the earliest mentions of the LHS as a concept only one decade ago [1, 2, 10]. This review has shown that the LHS can be a successful model to create effective bridges across silos of disciplines and professions, and facilitate the creative problem solving to solve complex problems that are often faced in healthcare to produce better health outcomes [67].

### Limitations

Limitations here include the heterogeneous nature of terminology used, the lack of structured descriptions of the LHS components, the varied outcomes and the need for narrative evidence synthesis. Also, only five (21.7%) LHS environments identified had produced medium-high level of evidence and all these were all in the United States. Another limitation of this review is that the majority of articles were identified following a grey-literature search of websites and other information; therefore, it is likely that there are other LHS environments that had reported impact, but used different terminology and were not captured in this review.

### Future research

Moving forward, common terminology is needed and core components of LHS need to be identified and reported, along with tangible healthcare impacts. Learning on both barriers and facilitators could also be better captured to advance the field. This review focused on high income countries only and the expansion of evidence and future updates of the review could include extending to low and middle income countries [68]. Additionally, the evidence from this review could be used to assist the development of LHS in high, mid and low income countries to enable better use of data to drive healthcare improvements and deliver impacts. Finally, the COVID-19 pandemic has resulted in rapid changes inside health systems globally, particularly as systems were adapted to conduct routine non-COVID healthcare remotely and to provide optimal treatments for patients with COVID-19. The crisis and transformation occurring in healthcare over the last 12 months, is deliberately not captured here and is the subject of a separate subsequent project.

## Conclusion

The wealth of currently available health data offers clear opportunities for health care improvement, however barriers to the capture, use and application of data are significant. The Learning Health System is emerging to take practice to data, data to new knowledge though analysis, knowledge to practice through translation. Here in this systematic review, we demonstrate that LHS across multiple continents and settings can generate measureable healthcare improvement. These LHS built on electronic medical records and/or linked data, clinical registers, community of practice networks, academic health science centre partnerships, medical collaboration or commercial operations. Key features include benchmarking and individual patient tracking longitudinally with outcomes readily available to patients, clinicians and health services at the point of care. Benefits included better patient self-management, improved clinician care, and optimised clinical service, organisation and/ or system-level performance and benefits to research. Core features of LHS included having strong partnerships, generating a shared vision across stakeholders, having agreed principles and governance, implemented systems and processes to enable iterative sustainable improvement, using longitudinal benchmarking. Key opportunities moving forward include harmonising terminology, capturing and sharing learnings on how to advance the LHS with greater research and evidence of translation into practice to deliver on the promise of health data to improve and transform healthcare.

## Supplementary Information

**Additional file 1.**

## Data Availability

The datasets used and/or analysed during the current study available from the corresponding author on reasonable request.
