# Supplementary material for: Learning health systems using data to drive healthcare improvement and impact: a systematic review
Source: BMC Health Serv Res. 2021 Mar 5;21:200. doi: 10.1186/s12913-021-06215-8 (PMC7932903; doi:10.1186/s12913-021-06215-8)
Supplement: Supplementary file 1 — Additional file 1. [file 12913_2021_6215_MOESM1_ESM.docx]

**APPENDIX 1 Search code used on the electronic databases**

A systematic search, based on the selection criteria and combining key words, was developed in conjunction with an expert librarian. The search strategy is limited to English language papers published in the previous five years.

Scopus:

( TITLE-ABS-KEY ( “learning health system*”) OR incubator* OR "incubator hub*" OR accelerator* OR "innovation hub* OR accelerator* OR" OR "data-driven" OR hub* ) ) AND ( health* OR ehealth ) AND ( partner* OR collaborat* OR "startup" ) AND ( data* OR informatic* OR infomatic* OR digital ) AND ( translation* ) AND ( LIMIT-TO ( PUBYEAR , 2019 ) OR LIMIT-TO ( PUBYEAR , 2018 ) OR LIMIT-TO ( PUBYEAR , 2017 ) OR LIMIT-TO ( PUBYEAR , 2016 ) OR LIMIT-TO ( PUBYEAR , 2015 ) OR LIMIT-TO ( PUBYEAR , 2014 ) ) AND ( LIMIT-TO ( LANGUAGE , "English" ) )

Medline:

KEY (“learning health system*”) OR incubator* OR "incubator hub*" OR accelerator* OR "innovation hub* OR accelerator* OR" OR "data-driven" OR hub* ) AND ( health* OR ehealth ) AND ( partner* OR collaborat* OR translation* OR "startup" ) AND ( data* OR informatic* OR infomatic* OR digital ) AND (limit to (yr="2014 -Current" and english and last 5 years))

Medline in-process (and other non-indexed citations):

Same as for Medline

Embase:

Same as for Medline

CINAHL Plus:

TX ( (“learning health system*” OR incubator* OR "incubator hub*" OR accelerator* OR "innovation hub* OR accelerator* OR" OR "data-driven" OR hub* ) ) AND TX ( ( health* OR ehealth ) ) AND TX ( ( partner* OR collaborat* OR "startup" ) ) AND TX ( ( data* OR informatic* OR infomatic* OR digital ) ) AND ( translation* ) )

Limiters - Publication Year: 2014-2019; English Language

Web of Science:

#11 AND #10

Indexes=SCI-EXPANDED, SSCI, CPCI-S, CPCI-SSH, ESCI Timespan=Last 5 years

# 11 (ALL=( translation* )) AND LANGUAGE: (English)

Indexes=SCI-EXPANDED, SSCI, CPCI-S, CPCI-SSH, ESCI Timespan=Last 5 years

#10: #9 AND #8 AND #7 AND #6

Indexes=SCI-EXPANDED, SSCI, CPCI-S, CPCI-SSH, ESCI Timespan=Last 5 years

# 9 (aLL=( data* OR informatic* OR infomatic* OR digital )) AND LANGUAGE: (English)

Indexes=SCI-EXPANDED, SSCI, CPCI-S, CPCI-SSH, ESCI Timespan=Last 5 years

# 8 (all=( partner* OR collaborat* OR "startup" )) AND LANGUAGE: (English)

Indexes=SCI-EXPANDED, SSCI, CPCI-S, CPCI-SSH, ESCI Timespan=Last 5 years

# 7 (ALL=( health* OR ehealth )) AND LANGUAGE: (English)

Indexes=SCI-EXPANDED, SSCI, CPCI-S, CPCI-SSH, ESCI Timespan=Last 5 years

# 6 ((ALL= (“learning health system*” OR incubator* OR "incubator hub*" OR accelerator* OR "innovation hub* OR accelerator* OR" OR "data-driven" OR hub* ))) AND LANGUAGE: (English)

Indexes=SCI-EXPANDED, SSCI, CPCI-S, CPCI-SSH, ESCI Timespan=Last 5 years

**APPENDIX 2 Template used to decide if studies were eligible.**

| EXCLUDE if one or more boxes ticked | Notes |
| --- | --- |
| Review article |  |
| Post hoc analyses of registry or survey data |  |
| Animal research |  |
| Poster abstract |  |
| Specific basic research (Lab-based); although systems and collaborative research maybe included |  |
| Non-English language article |  |
| Published before 2014 |  |
| Non-high income country |  |

| To pass the screen, at least one box is checked in each section  (If unsure, then retain for full text examination) | Notes |
| --- | --- |
| Includes Health managers, Healthcare providers | Participants (P) |
| New data capability embedded in health service to drive mobilisation and utilisation of data for the purpose of healthcare improvement  Data Healthcare improvement concept development  Embedded data role  Knowledge mobilisation or brokering (with data as significant component)  Data capability – how to use existing data  Using live data– frontline staff, data feedback | Intervention (I) |
| Impact on patients (pt reported experience)  Patient reported outcome measures  Utilisation of best practice guidelines  Access to integrated service systems utilising data  Evidence of translation into practice | Outcome (O) |
